# AutoDeconJ: a GPU-accelerated ImageJ plugin for 3D light-field deconvolution with optimal iteration numbers predicting

**DOI:** 10.1093/bioinformatics/btac760

**Published:** 2022-11-28

**Authors:** Changqing Su, Yuhan Gao, You Zhou, Yaoqi Sun, Chenggang Yan, Haibing Yin, Bo Xiong

**Affiliations:** School of Mechanical, Electrical & Information Engineering, Shandong University, Weihai 264209, China; National Engineering Laboratory for Video Technology (NELVT), Peking University, Beijing 100871, China; Lishui Institute of Hangzhou Dianzi University, Hangzhou 323000, China; School of Electronic Science and Engineering, Nanjing University, Nanjing 210023, China; Lishui Institute of Hangzhou Dianzi University, Hangzhou 323000, China; School of Mechanical, Electrical & Information Engineering, Shandong University, Weihai 264209, China; Lishui Institute of Hangzhou Dianzi University, Hangzhou 323000, China; Lishui Institute of Hangzhou Dianzi University, Hangzhou 323000, China; National Engineering Laboratory for Video Technology (NELVT), Peking University, Beijing 100871, China

## Abstract

**Motivation:**

Light-field microscopy (LFM) is a compact solution to high-speed 3D fluorescence imaging. Usually, we need to do 3D deconvolution to the captured raw data. Although there are deep neural network methods that can accelerate the reconstruction process, the model is not universally applicable for all system parameters. Here, we develop AutoDeconJ, a GPU-accelerated ImageJ plugin for 4.4× faster and more accurate deconvolution of LFM data. We further propose an image quality metric for the deconvolution process, aiding in automatically determining the optimal number of iterations with higher reconstruction accuracy and fewer artifacts.

**Results:**

Our proposed method outperforms state-of-the-art light-field deconvolution methods in reconstruction time and optimal iteration numbers prediction capability. It shows better universality of different light-field point spread function (PSF) parameters than the deep learning method. The fast, accurate and general reconstruction performance for different PSF parameters suggests its potential for mass 3D reconstruction of LFM data.

**Availability and implementation:**

The codes, the documentation and example data are available on an open source at: https://github.com/Onetism/AutoDeconJ.git.

**Supplementary information:**

Supplementary data are available at *Bioinformatics* online.

## 1 Introduction

The wonder of life’s activity lies in its ability to coordinate all its cells and tissues in an elegantly compact system to carry out its functions in an orderly manner. To get a glimpse of this mystery and explore the interrelationships between these parts, various imaging techniques have been proposed gradually, such as two-photon microscopy (Albota *et al.*, 1998), plane illumination methods (Huisken *et al.*, 2004) and confocal microscopy (Schulz *et al.*, 2013), allowing high spatial resolution 3D imaging (Planchon *et al.*, 2011; Wu *et al.*, 2021; Xiong *et al.*, 2021; Yang *et al.*, 2017). However, much of the interaction between cells and tissues occurs transiently in three dimensions (Prevedel *et al.*, 2014), perhaps in milliseconds or even microseconds. It requires imaging systems with the high spatiotemporal resolution, but the trade-off between space and time can hardly be effectively addressed. Most of the existing techniques prefer to reconstruct a 3D volume by recording a certain number of 2D images (Keller *et al.*, 2008), which is equivalent to sacrificing temporal resolution for 3D spatial resolution.

Light-field microscopy (LFM) has been emerging as a crucial volumetric imaging method due to its ability to capture 3D information in a tomographic manner within a snapshot (Xiong *et al.*, 2021; Cohen *et al.*, 2014). In view of its excellent volumetric imaging speed (Wang *et al.*, 2021), it is exceptionally well suited for high-speed volumetric imaging. As a result, a growing number of biological and medical researchers have paid special attention to applying it in their fields of studies, such as whole-animal 3D imaging of neuronal activity (Prevedel *et al.*, 2014), 3D behavioral phenotyping (Shaw *et al.*, 2018) and high-speed volumetric brain imaging (Zhang *et al.*, 2021). Despite these advantages having led to the rapid development of applications, the presence of post-processing steps for light-field images and the low throughput of the reconstruction algorithm at this stage limit its application for long-timescale real-time observation (Wang *et al.*, 2021). 3D Richardson–Lucy (RL) deconvolution algorithm has been widely applied to enhance the resolution of LFM, and Prevedel *et al.* (2014) have provided software for 3D volume reconstruction in MATLAB based on RL deconvolution (Prevedel *et al.*, 2014). The subsequent related deconvolution methods are also implemented based on RL deconvolution, such as phase-space deconvolution (Lu *et al.*, 2019) and high-resolution LFM (Li *et al.*, 2019). However, the reconstruction speed of these deconvolution methods is relatively slow, not enough for real-time observation. Although Prevedel *et al.* (2014) have introduced the GPU acceleration to the deconvolution of LFM data, it is only adopted in part of the convolution operation, thus limiting the overall acceleration performance. Moreover, it is inconvenient to utilize multiple GPUs simultaneously in MATLAB, which will limit its extension to large-size inputs due to the memory size of the image processor unit (GPU). The deep network XLFMNet (Vizcaino *et al.*, 2021) and VCD-Net (Wang *et al.*, 2021) have been proposed to boost the reconstruction throughput to a fantastic level. However, LFM data with different point spread function (PSF) parameters require training separate specific networks, which makes it trouble for biological researchers since biological observation usually requires different objectives. On the other hand, the trained network is only able to reconstruct the same type of data as the training data. Similarly, the image size in deep learning-based reconstruction is also limited by the memory of the GPU.

Here, to ensure the generality for different system parameters and convenience for users, we design AutoDeconJ, a plugin in ImageJ (Schindelin *et al.*, 2012) for 4.4× faster compared to the Matlab GUI program and accurate deconvolution of LFM data, improving both computational efficiency and convenience of interface interaction. We also add a module to measure the iteration result, predicting the optimal number of iterations. All the main functions of MATLAB versions (Prevedel *et al.*, 2014) are integrated into AutoDeconJ and optimized to take advantage of the parallel processing capacity on the GPU. We first put the time-consuming part of the computation on the GPU, including the part of PSF computing and deconvolution. To maximize the efficiency of parallel computing and solve the problem of insufficient memory on GPU, we also introduce a multi-GPU framework, in which the PSF computation and the reconstruction process of different axial layers in 3D imaging can be evenly distributed to different GPUs, thus doubling the throughput directly. The reconstruction speed is proportional to the number of GPUs in use theoretically. The RL deconvolution algorithm is an iterative process where the number of stop iterations is usually determined by empirical values (Lu *et al.*, 2019; Prevedel *et al.*, 2014). As the input data change, the empirical value may also change, which is highly inconvenient for the reconstruction of large amounts of different light-field data. Hence, we introduce a prediction module that can predict the optimal number of iterations based on the intermediate iterative results. ImageJ is a cross-platform application widely used in biological research (Schindelin *et al.*, 2012), and we thus choose it as our basis in the form of a plugin for all researchers to facilitate their use. We verify the ability of AutoDeconJ in the light-field fluorescence data of *Caenorhabditis elegans*, which comes from an open-source dataset (Prevedel *et al.*, 2014). Our developed AutoDeconJ shows its excellent facilitation to light-field reconstruction, including the large data throughput and accurate prediction for iterations. To further demonstrate the performance of AutoDeconJ, we test it on the fluorescence beads data and MCF10A cells data.

## 2 Materials and methods

### 2.1 GPU acceleration

**Fig. 1.**
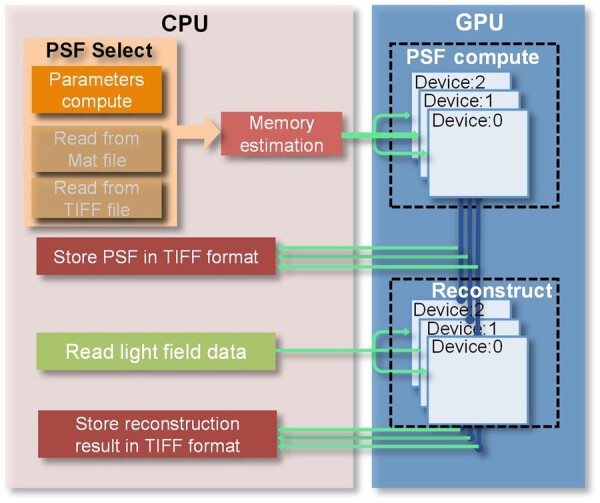
The acceleration framework of AutoDeconJ. AutoDeconJ puts the main time-consuming parts, including PSF computation and light-field reconstruction on the GPUs. First, the source of PSF needs to be selected in three ways: computing by specific parameters, reading from a MAT file or reading from a TIFF file. The default is computing by specific parameters. And then, AutoDeconJ will estimate the required memory size to facilitate the following memory allocation for multi-card operation. Whether the PSF is read from a file or calculated based on parameters, the PSF is evenly distributed to each card according to the memory size, which is used directly for the next reconstruction to reduce the time consumption of data transfer between GPU and CPU

The difference between AutoDeconJ and the previously released MATLAB GUI program (Prevedel *et al.*, 2014) is that AutoDeconJ takes full advantage of the highly parallel computation of the GPU. In the MATLAB GUI program, the calculation of the PSF is all performed on the CPU, which needs a substantial amount of computation. Specifically, the computation time in a single computational cycle is more than millions of microseconds which makes this entire step very time-consuming on the CPU serial data processing model. AutoDeconJ has two significant improvements compared to the MATLAB GUI program. The first improvement is in the acceleration of the calculation of PSF. AutoDeconJ transfers the main time-consuming parts in PSF calculating to the GPU. The speed of PSF calculation can be improved by exploiting the parallel computation (e.g. it achieves more than 20 times improvement in the experiment of *C.elegans*). Another one is our proposed strategy of data processing which is shown in Figure 1. AutoDeconJ puts most of the operations to GPU during the deconvolution process, and only transfers the final results back to the CPU, avoiding transferring the intermediate results frequently between CPU and GPU, which is time-consuming. In addition, AutoDeconJ also provides support for multi-GPU collaboration to cope with the problem of insufficient memory. After all, not every researcher can afford expensive professional GPUs with large memory. We divide the 3D layers to be reconstructed evenly among different GPUs according to the number of GPUs so that we can handle large-size reconstructions and increase the reconstruction speed exponentially.

### 2.2 Image quality metric

In the field of image processing, the discrete cosine transform (DCT) has been widely applied to transfer spatial information to the spectral domains, because of its great de-correlation and lossless property (Kristan *et al.*, 2006). The most common mathematical method is to project an image onto an orthonormal basis in which the amplitudes are called the DCT coefficients. Compared to the discrete Fourier transform, the obvious advantage of the DCT is that its coefficients are only represented by real numbers without the complicated complex number operations (Blinn, 1993). Its computing efficiency thus can be significantly improved, making it applicable to some real-time systems. The DCT coefficient transformed from an image function f(x,y) can be obtained via Eq. (1).

(1) $$F_{c}(u,v)=\sum_{\left(x,y\right)}f(x,y)C_{u}(x,M)C_{v}(y,N),$$

where x∈{0,…,N−1}, y∈{0,…,M−1}, M is the number of pixels along the height, and N is the number of pixels along the width in the image. $C_{u}(x,M)C_{v}(y,N)$ is the basis functions defined by the following expressions:

(2) $$C_{u}(x,M)=c(u,M)*\;\text{cos}(\frac{(2x+1)\pi u}{2M}),$$

(3) $$C_{v}(y,N)=c(v,N)*\;\text{cos}(\frac{(2y+1)\pi v}{2N}),$$

(4) $$c(\varphi ,Z)=\left\{ \begin{array}{c} \frac{1}{\sqrt{Z}};\varphi =0,\\ \sqrt{\frac{2}{Z}};\text{otherwise}. \end{array}\right.$$

**Fig. 2.**
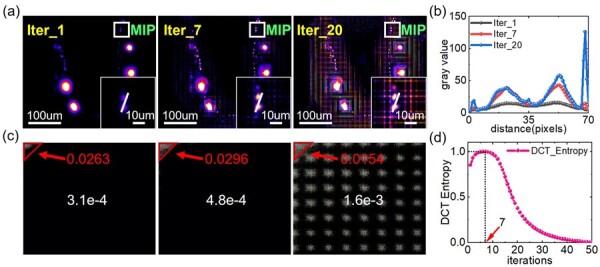
Performance of the image quality metric. The source light-field data is obtained from the open-source dataset provided in Prevedel *et al.* (2014). (**a**) The maximum projection along the z-axis for each result with the RL deconvolution at 1, 7 and 20 iterations. The image in the lower-right corner is a magnification of the white area. (**b**) The gray value along solid lines in the lower-right corner of the diagram in (a). (**c**) The DCT transform corresponding to the deconvolution result in (a). The coefficients with higher values are shown in light gray, and those with lower values are shown in dark gray. The red numbers are the DCT entropy values of the red triangle in the upper left corner whose size is determined by Eq. (6), and the white numbers indicate the overall DCT entropy value. By visual comparison of the red region as well as the values, it can be observed that with the increase of the iterations, the entropy value in the low-frequency region circled in red will increase first and then become smaller again but the overall entropy keeps rising. (**d**) Normalized curve of DCT entropy values with the number of iterations for the red region in (c), where the maximum DCT entropy value corresponds to the number of iterations of 7, which is consistent with the empirical value

In general, microscopic observations are made to obtain as much high frequency information as possible. However, due to the limitations of the acquisition system, the resolution information it can obtain is usually in the range of a certain cut-off frequency. We usually pay more attention to information close to the cut-off frequency in the cut-off frequency range rather than near direct current. The relationship between the number of iterations and the image spectrum is illustrated in Figure 2a and c, where the source light-field image is obtained from the open-source dataset provided in Prevedel *et al.* (2014). We only take the maximum projection along the z-axis for each iterative result with RL deconvolution.

The deconvolution of the light-filed images can be considered a process of rearrangement of the aliased signal in the low- and high-frequency regions, which is reflected in the energy increase in the cut-off frequency range and the extension of effective spectral range in the spectrum of the image. Even the effective spectral range of the reconstructed results will exceed the original cut-off frequency to some extent, due to the additional information introduced by the PSF. Proper deconvolution operations can restore the high-frequency detail while maintaining low-frequency features. However, excessive deconvolution operations will destroy the original structural information of the image, which is reflected in the DCT-transformed image with the periodic spectrum shift, as it has shown in Figure 2a–c. During the process of iteration, the maximum amount of information will be presented in and around the cut-off frequency range when the information has been restored to the best. Since the recovery of 3D information is limited, there should be a boundary to the effective spectral region whose size is related to the maximum resolution the optical system can obtain, as shown in Figure 2c. A standard metric to measure the region with uniform distribution is the Shannon entropy (Kristan *et al.*, 2006), defined as

(5) $$F_{\mathrm{entropy}}=-\sum_{i\in S}p_{i}{\;\text{log}\;}_{2}(p_{i}),$$

where $p_{i}$ is a probability function defined on the region S. Theoretically, we should calculate the entropy of the region area bounded by an arc in the upper left corner, since the region is determined by the cut-off frequency which usually is a quarter circle or ellipse. The cut-off frequency could be calculated from the resolution limit of the system. The entropy of the region area bounded by an arc in the upper left corner represents the amount of information collected by the system within its own limit resolution. The entropy increase during the iteration means the reconstruction algorithm restores more valid information. However, in order to reduce the computational consumption, S is selected as a triangular region in the upper left corner in DCT, whose size is defined as

(6) $$G_{S}=X_{S}*Y_{S}/2,$$

where $X_{S}$ is the number of pixels along the width, and $Y_{S}$ is the number of pixels along the height in the region S.

For the DCT of an image, assuming that the pixel size is $P_{u}$, then the spectral range corresponding to the DCT image is $\left[0,\frac{1}{2P_{u}}\right]$. Assuming that the limit of the resolution for a wide-field microscope is $d_{\mathrm{psf}}$, and its corresponding frequency position P in the DCT image can be defined by:

(7) PW=12dpsf12Pu

(8) $$P=\frac{P_{u}}{d_{\mathrm{psf}}}W$$

where W is the pixel number of a dimension (such as M and N in Eq. (1)) in the DCT image. According to the Eq. (8), $X_{S}$ and $Y_{S}$ can be obtained by:

(9) $$X_{S}=P_{u}*N/d_{\mathrm{psf}}$$

(10) $$Y_{S}=P_{u}*M/d_{\mathrm{psf}}$$

It is well known that we can estimate the size of the Airy spot from the diffraction theory. For the objective lens, the size of one Airy unit is $\frac{1.22\lambda }{NA}$ (Wang *et al.*, 2010), where NA is the numerical aperture of the objective lens and λ is the wavelength of emission light. The above-mentioned $d_{\mathrm{psf}}$ refers to the smallest resolvable distance between two Airy spots. $d_{\mathrm{psf}}$ can be determined by

(11) $$d_{\mathrm{psf}}=\frac{0.61\lambda }{NA}$$

However, LFM sacrifices spatial resolution to capture additional angular information. Here, assuming that *N*_num_ is the virtual pixels for each microlens, which also represents the angular resolution. From the DCT image of the 20th iteration of *C.elegans* data (*N*_num_ = 15), as shown in Figure 2c, it can be found that the spectral period in the DCT image is only half of the *N*_num_. As such, in LFM, assuming that $P_{u\_LF}$ is the pixel size of light-field microscopy data, its corresponding frequency position $P_{LF}$ in the DCT image should be:

(12) $$P_{LF}=\frac{P_{u\_LF}}{d_{\mathrm{psf}}}W/\left(\frac{N\text{num}}{2}\right)$$

and the pixel size $P_{u\_LF}$ can be defined as:

(13) $$P_{u\_LF}=\frac{d_{ML}}{Q*N\text{num}}$$

where $d_{ML}$ is the microlens pitch size, and Q is the magnification of the objective lens. In the LFM, assuming that $X_{S\_LF}$ is the number of pixels along the width of S and $Y_{S\_LF}$ is the number of pixels along the height of S. According to the Eq. (12), $X_{S\_LF}$ and $Y_{S\_LF}$ can be obtained by:

(14) $$X_{S\_LF}=2*P_{u\_LF}*N/\left(N\text{num}*d_{\mathrm{psf}}\right),$$

(15) $$Y_{S\_LF}=2*P_{u\_LF}*M/(N\text{num}*d_{\mathrm{psf}})$$

The final image metric expression, called DCT entropy, is as follows:

(16) $$DCT_{entropy}\overset{\;}{=}-\frac{2}{S^{2}}\sum_{\begin{array}{l} u<X_{S\_LF}\\ v<Y_{S\_LF} \end{array}}\left|\frac{F_{c}(u,v)}{L_{2}\left(F_{c}(u,v)\right)}\right|{\mathrm{abslog}}_{2}\left(\frac{F_{c}(u,v)}{L_{2}\left(F_{c}(u,v)\right)}\right)$$

where $L_{2}(•)$ is the 2-norm of a matrix and $F_{c}(*)$ is DCT which can be referred to Eq. (1). As for DCT entropy calculation in the wide-field microscopy, the range of u is determined by Eq. (9) and the range of v is determined by Eq. (10), while others remain the same. As in Figure 2d, we calculate the DCT entropy in the region limited by the optical resolution of reconstructed images with different iteration numbers (1–50) and plot the normalized curves accordingly. The results show that the entropy maximum is at the results of the seventh iteration, which is consistent with the empirical value. In practice, we can stop iteration when the DCT entropy value shows a decreasing trend. In order to demonstrate the calculation of DCT entropy must be restricted in the limited region, we also calculate the DCT entropy of the whole DCT (WDCT) spectrum of the x–y MIP images with the corresponding iterative numbers shown in Figure 2c, which is marked by the white numbers and named WDCT entropy. As the number of iterations increases, the WDCT keeps rising while the quality of the reconstruction does not. We further demonstrate the performance of our image quality metric on data acquired with standard wide-field microscopy, which shows good performance on iteration number prediction with the RL, RL-TV and landweber algorithms. More details can be found in the Supplementary materials.

## 3 Results

Here, we present AutoDeconJ, an ImageJ plugin with a GPU acceleration framework and iterative prediction module for light-field deconvolution. Compared to the specificity and the complicated preparation process of reconstruction by deep learning, the main improvement of AutoDeconJ is to provide a universal tool for light-field reconstruction with a decent data throughput, a friendly interactive interface and the potential to scale to large input sizes. AutoDeconJ is convenient for the user without a background in computer science. In addition, the introduction of the novel iterative criterion in AutoDeconJ can further enhance the user’s ability to cope with different kinds of input data.

### 3.1 Availability

Benefiting from ImageJ with powerful cross-platform capability, AutoDeconJ can run on any system with ImageJ or Fiji installed. For details required for ImageJ installation, see the official website *imagej.net* for more information. AutoDeconJ requires the NVIDIA cards support by CUDA8.0 or later. See https://developer.nvidia.com*f*or more details about CUDA. If AutoDeconJ needs to run under multiple NVIDIA cards, please ensure that the system is equipped with multiple NVIDIA cards. Our recommendation for these cards is to support the scalable link interface or NVLINK, which can further enhance the reconstruction speed. For a detailed user manual, please see supplementary materials. The ImageJ plugin source code is already available on https://github.com/Onetism/AutoDeconJ.git. Please clone it to the local folder, then follow the tutorial to compile it and move the jar package to the/*plugin*/folder (where ImageJ is installed) to complete the installation.

### 3.2 Comparison

During development, the main target was to design a universal tool. As such, a final comparison of AutoDeconJ to the Matlab GUI program was performed using the datasets provided in Prevedel *et al.* (2014), including the fluorescence beads data and MCF10A cells data. In summary, AutoDeconJ performs as well as the Matlab GUI program in terms of reconstruction quality but requires less time-consuming. It also provides a more friendly interactive interface and a better data throughput. Furthermore, the optimal iteration number predicted by our proposed prediction module is consistent with empirical values, which can be used as a reference for the iterative reconstruction of new light-field data. Specific details of the comparison are provided in the Supplementary materials. In the end, we also demonstrate the poor data migration capability of the state-of-the-art VCD-Net network on simulated data, which is also presented in the Supplementary materials.

## Supplementary Material

btac760_Supplementary_Data

## Data Availability

The example data for C. elegans in figure 2 are available at https://static-content.springer.com/esm/art%3A10.1038%2Fnmeth.2964/MediaObjects/41592_2014_BFnmeth2964_MOESM189_ESM.zip and the data in supplementary material are available from the corresponding authors upon reasonable request.
