# Supplementary material for: AutoDeconJ: a GPU-accelerated ImageJ plugin for 3D light-field deconvolution with optimal iteration numbers predicting
Source: Bioinformatics. 2022 Nov 28;39(1):btac760. doi: 10.1093/bioinformatics/btac760 (PMC9805591; doi:10.1093/bioinformatics/btac760)
Supplement: btac760_Supplementary_Data [file btac760_supplementary_data.zip › btac760_Supplementary_Data/AutoDeconJ_user_manual.docx]

# Setup

## 1.1 Requirements

## 1.1.1 ImageJ

AutoDeconJ requires the desktop to have ImageJ2.01 (http://imagej.net/) or Fiji2 (https://fiji.sc/) installed. AudoDeconJ needs to have writing access to the configure file in ImageJ2.0 or Fiji. The following ImageJ defaults refer to ImageJ2.0.

## 1.1.2 OS requirements

Any system capable of running ImageJ or Fiji can run AutoDeconJ. For more details, please refer to the official guideline of ImageJ (<http://imagej.net/Downloads>), where a brief system requirement description includes：

ImageJ will run on any system that has a Java 8 (or later) runtime installed. This includes, but is not limited to:

1. Windows XP, Vista, 7, or 8 with Java installed from java.com

2. Mac OS X 10.8 “Mountain Lion” or later with Java installed from java.com

3. Ubuntu Linux 12.04 LTS or later with OpenJDK 8 installed

The calculation of the point spread function and the reconstruction of light field images require large memory. As such, if conditions permit, set the heap memory in Java as large as possible to ensure that AutoDeconJ can cope with large-scale inputs. Java heap space memory is set in */Edit/Options/Memory & Threads* (see fig. 1).


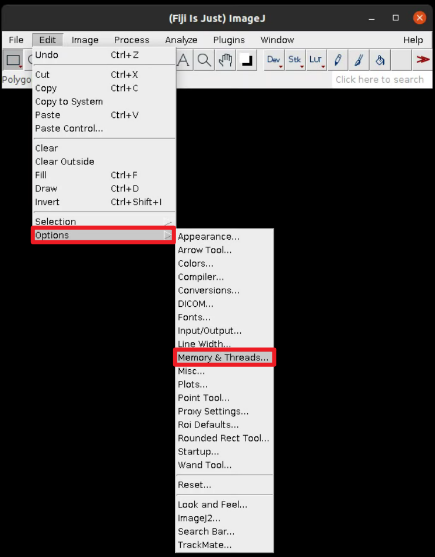


*Figure 1 Fiji and Memory & Threads*

As far as we know, NVIDA no longer provides update for Cuda 10.2 for Mac OS, and Apple has started to stop supporting NVIDA CUDA in Mac OS Mojave. Therefore, the AutoDeconJ execution in these cases is not stable. For more details, please refer to [www.apple.com](http://www.apple.com) and www.nvidia.com.

## 1.1.3 Hardware requirements

AutoDeconJ requires an available NVIDIA card that is supported by cuda8.0 or later, since the AutoDeconJ performs time-consuming processes on the system's available GPU. If a compatible NVIDIA card is not available, AutoDeconJ will not run properly and be interrupted early. Before all these, make sure that the latest drivers for NVIDIA card are installed. The more memory the NVIDIA cards have, the larger the input image size AutoDeconJ can support.

In addition to increasing the memory of a single NVIDIA card, it is also available to connect multiple cards with small memory together to support larger input sizes. If these connected cards are supported by Scalable Link Interface (SLI) (e.g., TiTan Xp, GTX1080Ti) or NVLink (e.g., Tesla V100, Tesla A100), AutoDeconJ can have greater improvement. For more details, please refer to the official instructions of NVIDIA (<https://www.nvidia.cn/geforce/technologies/sli/> or <https://www.nvidia.cn/data-center/nvlink/> ).

## 1.1.4 JCuda3

AutoDeconJ requires OS-specific JCuda archives installed in ImageJ or Fiji. Download the Jcuda-specific .jar file that matches the system and move it into \plugins\ directory where the ImageJ or Fiji install. For the latest OS-specific JCuda version installed, the source code of JCuda will need to be downloaded and recompiled. A suggested approach for installing JCuda is to download it as a direct package via apache maven. By default, JCuda archives are contained in AutoDeconJ installing package, and there is no need to install JCuda separately here.

## 1.1.5 Maven

AutoDeconJ's project is managed by Maven, so compiling AutoDeconJ requires that Maven is installed on the system. For details of maven installation, please refer to the official website (<https://maven.apache.org/install.html>).

## 1.1.6 Git

The source code of AutoDeconJ is managed by Git. As such, cloning the source code of AutoDeconJ requires the Git have been installed on the system. For details of git installation, please refer to the official website (<https://git-scm.com/downloads>). And of course, the zip file of AutoDeconJ can also be downloaded from GitHub directly instead of using Git.

## 1.1.7 Java 8

AutoDeconJ is written in Java and ImageJ only supports Java 8. Therefore, the system requires installing Java 8 and setting the proper environment variable of JAVA_HOME for maven building. For more details about java installation, please refer to <http://openjdk.java.net/> or <https://www.oracle.com/java/>.

## 1.2 Install AutoDeconJ

## 1.2.1 Cloning the repository

Create a local clone of the AutoDeconJ project in the terminal by calling

*git clone* [*https://github.com/Onetism/AutoDeconJ.git*](https://github.com/Onetism/AutoDeconJ.git)

or directly download the zip file from the Github repository.

## 1.2.2 Building the plugin jar

The cloned project can be opened and edited in any IDE (e.g., Eclipse, visual studio code, IntelliJ, and so on). However, the recommended way to build the final plugin JAR is using Apache Maven.

To build the plugin JAR, change it into the root directory of the project and execute

*mvn clean package*

Note that the resulting JAR file has a different name from the default name that Maven would assign to it. In order to properly be recognized as an ImageJ plugin JAR, it is named AutoDeconJ_Plugin-jar-with-dependencies.jar. Copy the resulting /target/ AutoDeconJ_Plugin-jar-with-dependencies.jar file into the \plugins directory of your Fiji or ImageJ installation. Of course, AutoDeconJ_Plugin-jar-with-dependencies.jar is also an execution file, and you can execute it directly.

## 1.2.3 Check AutoDeconJ installation

Restart ImageJ or Fiji and Check if *AutoDeconJ* is available under the *plugin* menu (see fig. 2). If not, please recheck that the previous steps have been completed correctly.


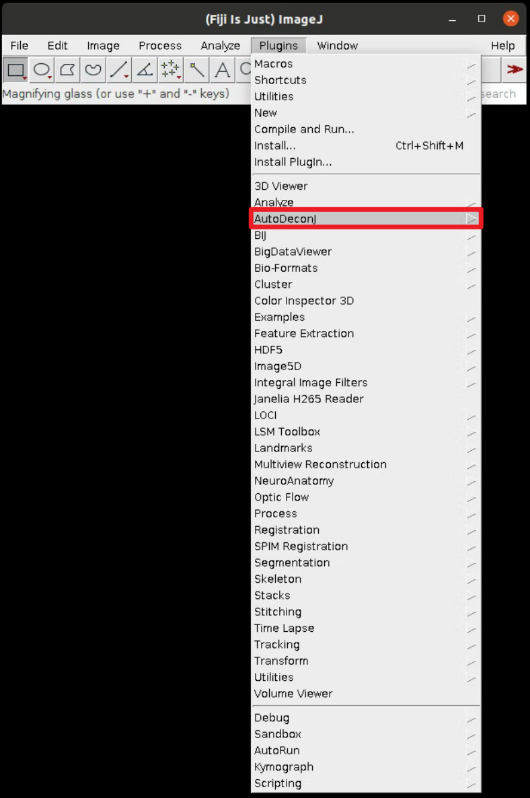


*Figure 2 Check AutoDeconJ installation*

# How to use

The following section will describe the proper use of the various module of AutoDeconJ, including the meaning of each parameter. AutoDeconJ provides two main functions (see fig. 3), one is named *ImageRectification*, which is mainly to realign the light field image, and the other is *Auto_LF_Deconvolution*, which is mainly to do the light field PSF calculation and light-field reconstruction. AutoDeconJ will use the last parameter as the default value.


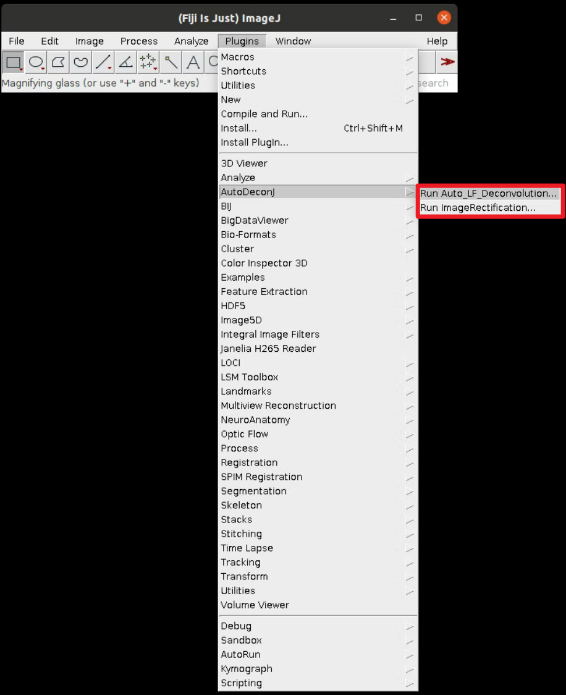


*Figure 3 Two main functions in AutoDeconJ*

**2.1 Image Rectification**

This module provides rectification of light field images by:

1. Loading the source light-field image into ImageJ.
2. Run *AutoDeconJ* /*ImageRectification*
3. *..*. from /plugins/ menu.
4. Setting the correct Rectification parameters.
5. Press the OK button.
6. Choose the storage path for the Rectification results.

The running phase will be displayed as a pop-up window and once completed. The corrected image will be visible under the selected storage path.

**2.1.1 Parameters Settings**


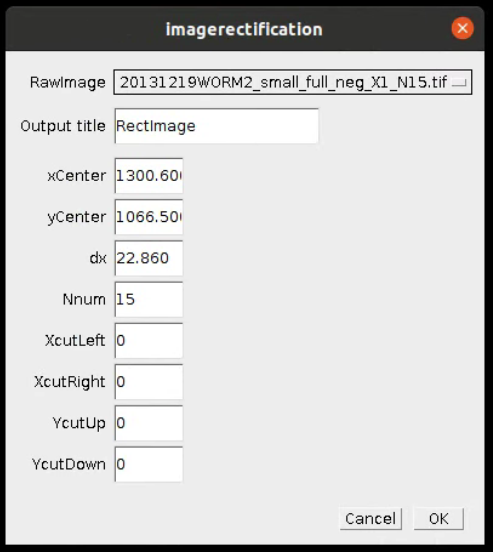


*Figure 4 Parameters settings in ImageRectification*

ImageRectification module requires the same import parameters settings (see fig. 4), taken from the reference [4]:

1. RawImage: If ImageJ has more than one image open, you can select the image to be rectified from the drop-down list, with the name corresponding to the window name of the opened image.
2. Output title: Specify the output file name.
3. xCenter: x-coordinate of the center point of one of the "light field circles".
4. yCenter: y-coordinate of the center point of one of the "light filed circles".
5. dx: distance between the "light field circles". This software assumes that the microlens array is perfectly aligned with respect to the pixel array (which is the image sensor) in terms of rotation.
6. Nnum: number of virtual pixels (either in x or y direction) under each microlens (i.e., there will be Nnum Nnum virtual pixels under each microlens). This has to be consistent with Nnum used for computing PSF.

The following parameters are used to limit the field of view in light field images:

1. XcutLeft: Specify the number of "light field circles" to be excluded at x-coordinate of starting position.
2. xCutRight: Specify the number of "light field circles" to be excluded at y-coordinate of end position.
3. YcutUp: Specify the number of "light field circles" to be excluded at y-coordinate of starting position.
4. YcutDown: Specify the number of "light field circles" to be excluded at y-coordinate of end position.

**2.2 Auto_LF_Deconvolution**


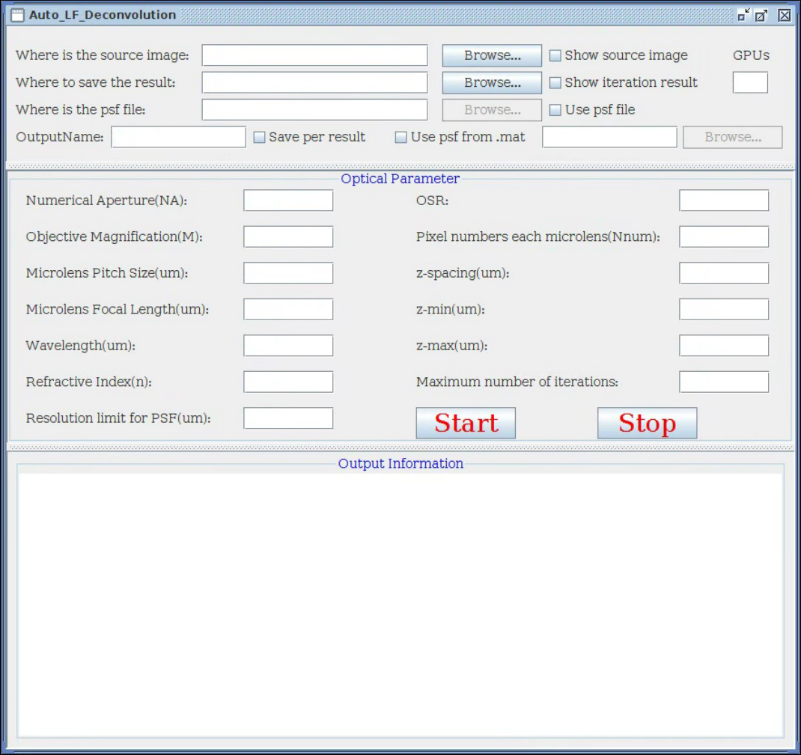


*Figure 5 Parameters settings in Auto_LF_Deconvolution*

This module provides two main functions: the calculation of the PSF and the reconstruction of the light field image (see fig. 5):

1. Run *AutoDeconJ*/*Auto_LF_Deconvolution...*. from /plugins/ menu.
2. Setting the correct parameters.
3. Press the *Start* button.

Intermediate information about the running phase will be printed in the Output Information box below. Pressing the *Stop* button can interrupt the service and close the ImageJ.

**2.2.1 Select the input and output**


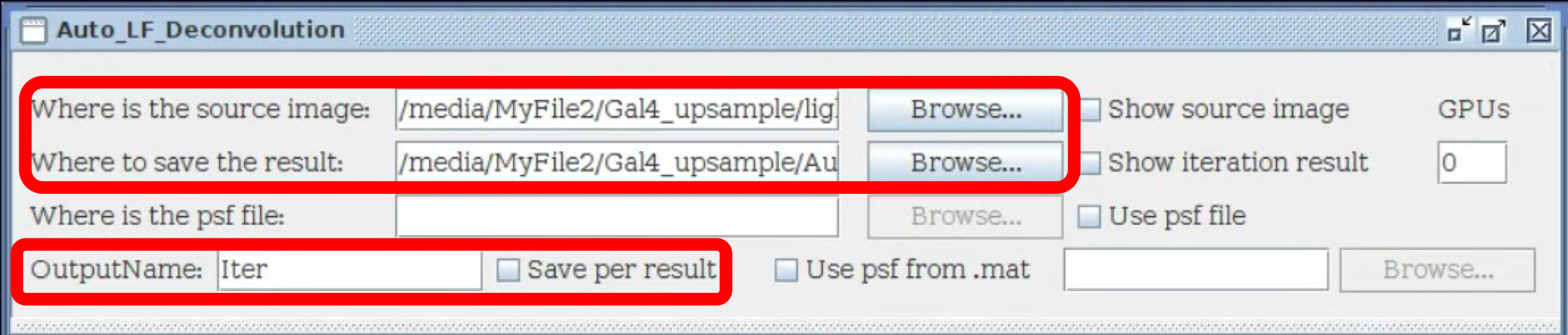


*Figure 6 Input and output settings*

Press the Browse button on the right side of the text "Where is the source image" to browse the path of the source image data. Please note that only the TIFF format files are supported here. Press the Browse button on the right side of the text "Where to save the result" to browse the path for saving the result. Once the path is selected correctly, the path will be shown on the box next to the button. The box next to the text "OutputName" is used to specify the name of the file to be saved. The check-box named "Save per result" is used to decide whether to save the results of each iteration (see fig. 6).

**2.2.2 PSF from Mat file**


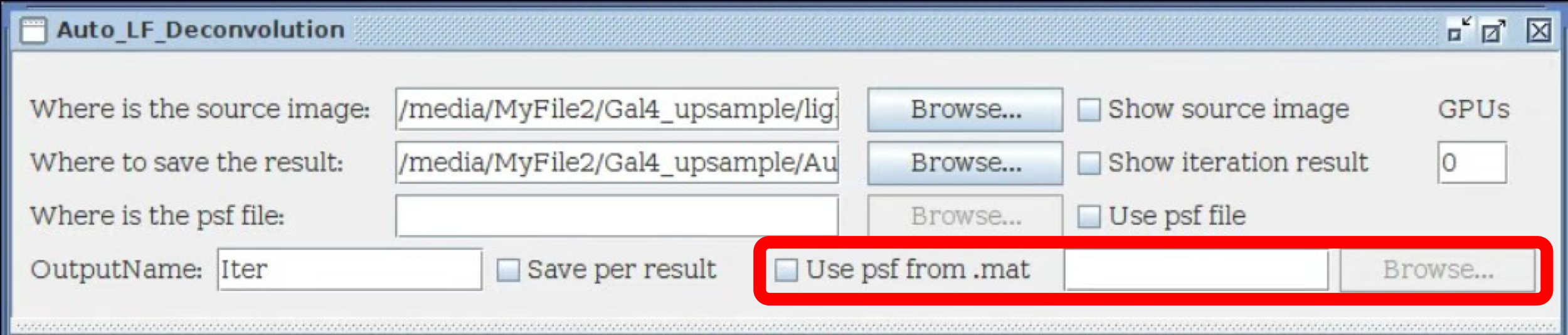


*Figure 7 Select PSF from the MAT file*

If there is a PSF file in .mat format, AutoDeconJ can directly load it, with the check-box named "Use psf from .mat" being selected and browsing the path where the PSF is located beside the check-box. Note that only the v7.0 version of the MAT file can be read successfully (see fig. 7).

**2.2.3 PSF from TIFF file**


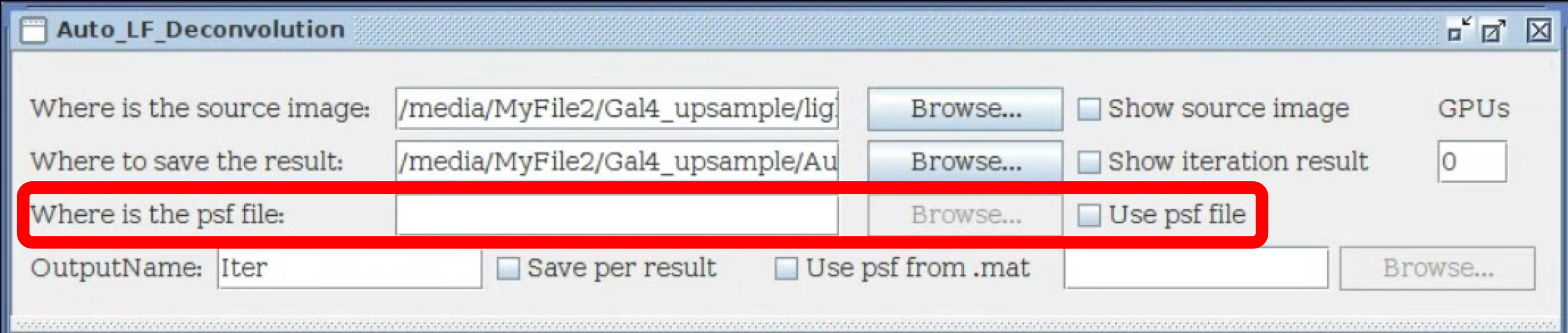


*Figure 8 Select PSF from the TIFF file*

If the PSF calculated by AutoDeconJ before in TIFF format is available, AutoDeconJ can also directly load it, with the check-box named "Use psf file" being selected and browsing the path where the PSF is located beside the check-box (see fig. 8).

**2.2.4 PSF calculated from specified parameters**

When the check-box named "Use psf file" and the check-box named "Use psf from .mat" are not selected, the PSF will be calculated according to the specified parameters (see fig. 9).


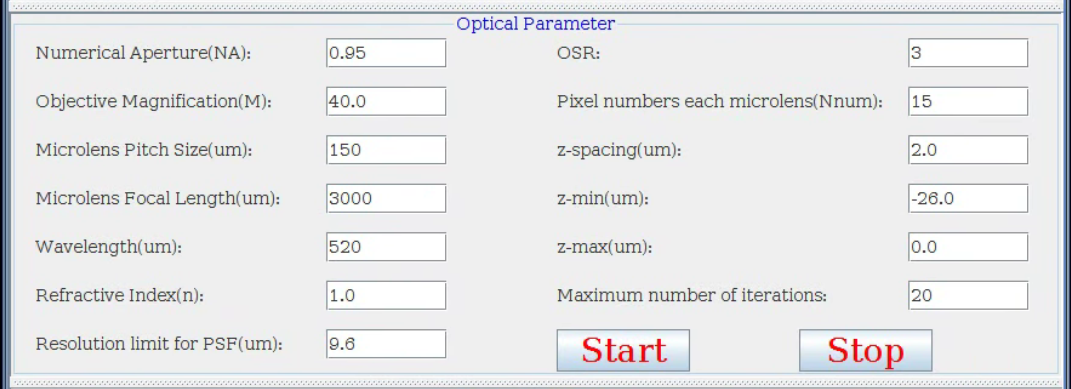


*Figure 9 Calculation of PSF based on specified parameters*

The panel of the Optical Parameter (see fig. 9) includes:

1. Numerical Aperture (NA): numerical aperture of the objective lens.
2. Objective Magnification (M): magnification of the objective lens.
3. Microlens Pitch Size (um): Pitch of the microlens array.
4. Microlens Focal Length (um): focal length of the microlens array.
5. Wavelength (um): wavelength of emission light.
6. Refractive Index (n): refractive index of the immersion material.
7. Resolution limit for PSF (um): the upper limit of the optical system resolution.
8. OSR: spatial oversampling ratio for computing PSF.
9. Pixel numbers each microlens (Nnum): number of virtual pixels (either in x or y direction) under each microlens (i.e., there will be Nnum Nnum virtual pixels under each microlens). This has to be consistent with Nnum used for rectifying the input image.
10. z-spacing (um): spacing between adjacent z-planes.
11. z-min (um): the axial location of the lowest z-­‐plane with respect to the focal plane. Larger value indicates a plane that is farther from the objective lens, where 0 corresponds to the exact focal plane.
12. z-max (um): the axial location of the highest z-plane with respect to the focal plane.
13. Maximum number of iterations: the upper limit of the number of times the iteration process can be executed, which is used to prevent infinite iterations.

Ensure that all the parameters are correctly specified, and then press the Start button to start running.

**2.2.5 GPU acceleration**


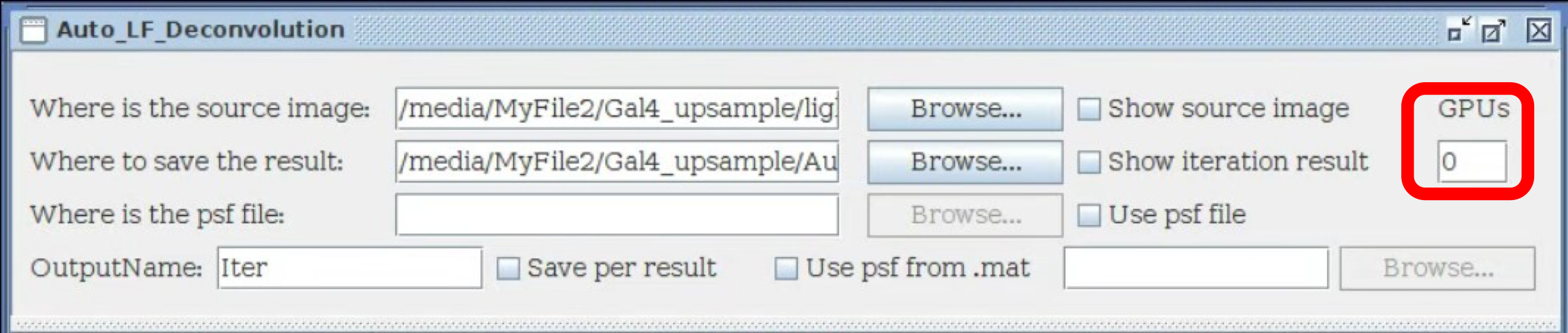


*Figure 10 Specify the number of GPUs to be used*

The input box in the upper right corner of the Auto_LF_Deconvolution panel, is used to specify the number of NVIDIA cards to be used (see fig. 10). The more cards are used, the less computing time will cost. For large input images, please estimate the required memory size in advance, and then make sure the number of NVIDIA cards is not less than a certain value (obtained by dividing the amount of required memory by the amount of memory on a single card). Refer to section 4.1 for estimated memory requirements.

**2.2.6 Display**


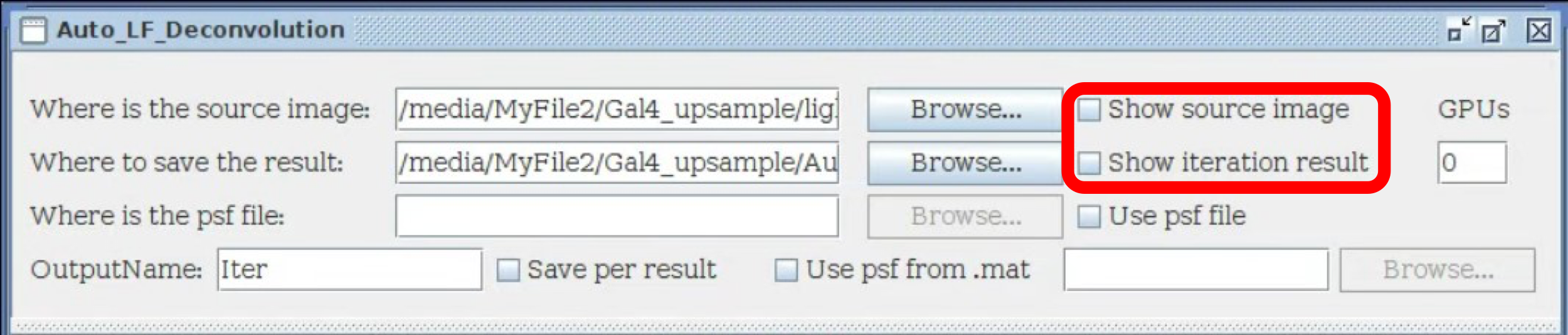


*Figure 11 AutoDeconJ display settings*

Auto_LF_Deconvolution provides two main display functions (see fig. 11). When the check-box named "Show source image" is selected, it will open a window showing the original light field image. When the check-box named "Show iteration result" is selected, the 3D results of each iteration will be displayed in a separate window. Additional run information will be printed in the box named "Output Information" (see fig. 12).


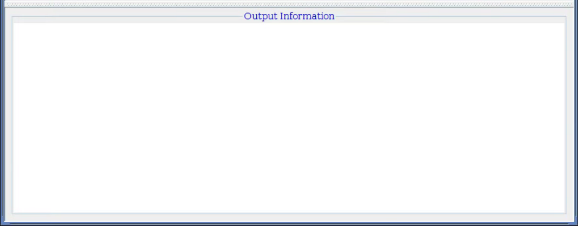


*Figure 12 The box for information output*

# Example

This section will use an example to illustrate the complete process of AutoDeconJ, including the rectification of light-filed microscopy data and the light-field reconstruction. The input data adopted in the example are the images of C.elegans provided by [4].

If the original light field data does not need rectification, please directly skip to Section 3.2.

**3.1** **Image Rectification**

In this section, the image named "20131219WORM2_small_full_neg_X1.tif" is loaded into ImageJ (see fig. 13).


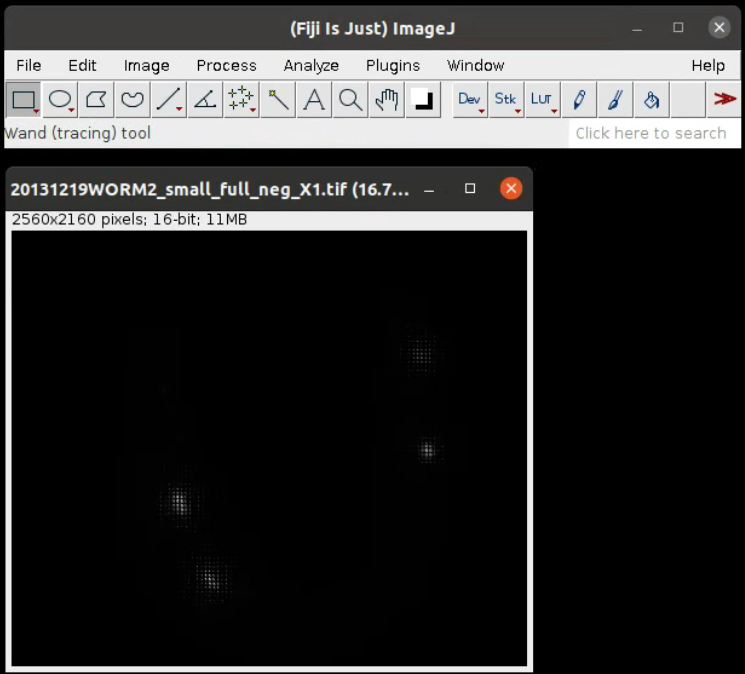


*Figure 13 The image is loaded into ImageJ*

The module of ImageRectification can be started (see fig. 14) by:

1. Loading the source light-field image into ImageJ.
2. Run AutoDeconJ from /plugins/ menu.
3. Select the *Run ImageRectification..*.


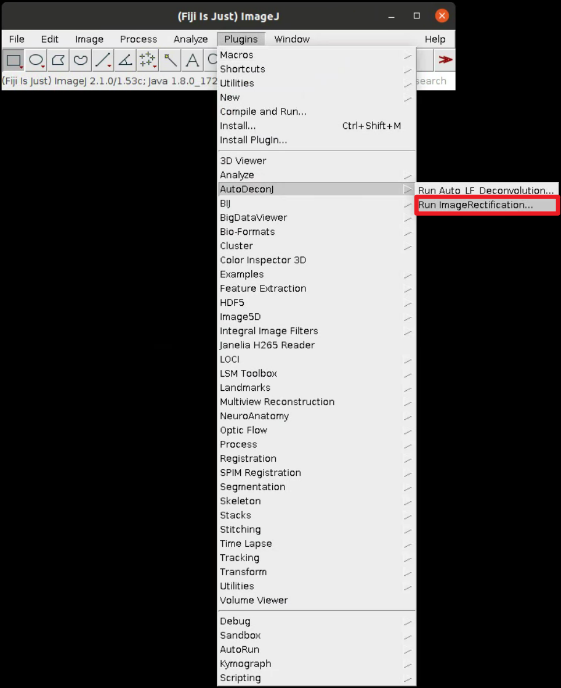


*Figure 14 Select ImageRectification function in AutoDeconJ*

The parameter setting window for rectification will pop up (see fig. 15).


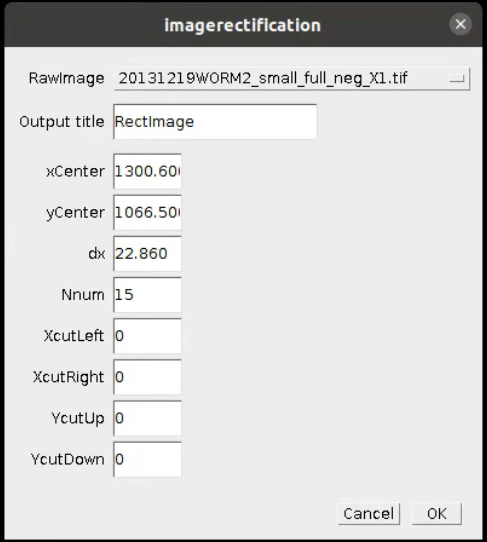


*Figure 15 Parameters setting window in ImageRectification*

The known coordinate position of the center of a microlens is (1300.6, 1066.5) pixels, the adjacent microlens pitch is 22.860 pixels, and the number of virtual pixels (either in x or y direction) under each microlens is 15. The field of view is not cropped by default. The "RawImage" is selected as the test image just opened, and the image output name is set as "RectImage". The parameter input is shown in figure 5. And then, ensure that all the parameters are set correctly and press the OK button. A new window will pop up, asking us to choose a path to store the results (see fig. 16).


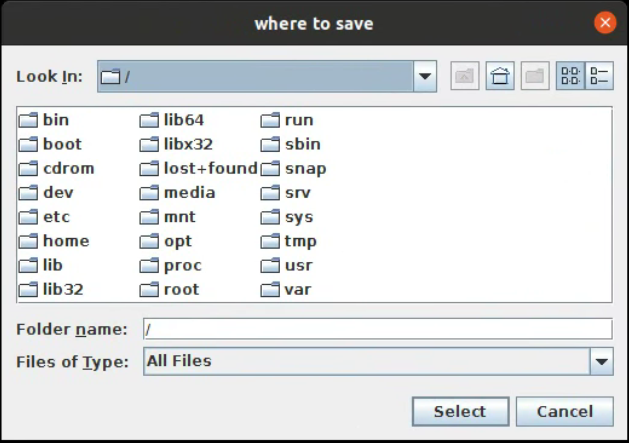


*Figure 16 Select path to store ImageRectification result*

For example, choose the root path here and click the "Select" button. AutoDeconJ will run in the background, and the intermediate information of the running process will be displayed under the toolbar of ImageJ. A new pop-up window named "RectImage" will appear showing the rectification results.


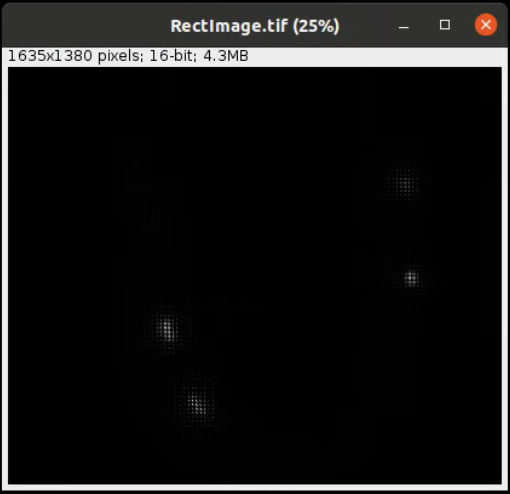


*Figure 17 The Image Rectification result*

**3.2 Auto_LF_Deconvolution**

In this section, the reconstruction of the light field image is performed (see fig. 18) by:

1. Run AutoDeconJ from /plugins/ menu.
2. Select the *Run Auto_LF_Deconvolution..*.


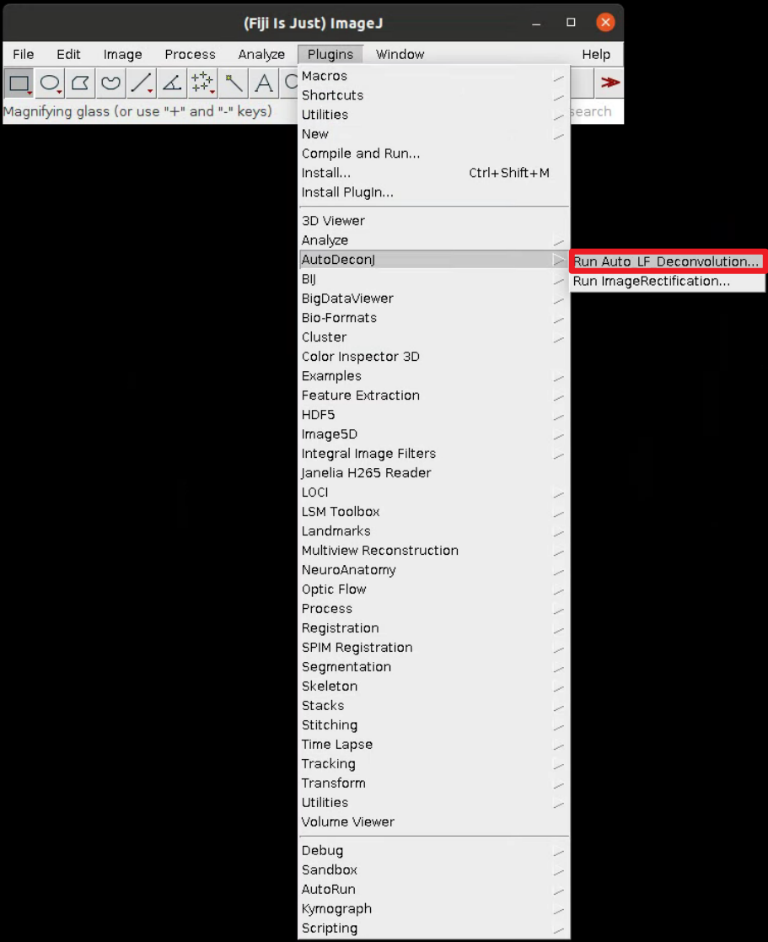


*Figure 18 Select Auto_LF_Deconvolution function in AutoDeconJ*

The parameter setting window for optical settings will pop up. We choose to obtain the PSF by numerical simulation based on optical parameters, which is provided in reference [4]. The source light field image is at the root path, and the output path for the result is set to /home. The parameters named " Resolution limit for PSF " is defined as

whereis the virtual pixels for each microlens, is the numerical aperture of the objective lens, and is the wavelength of emission light. Based on the example parameter, we can get the value of " Resolution limit for PSF " as 0.39. To speed up this progress, we choose to use two NVIDIA cards. The final parameter settings are shown in figure 19.


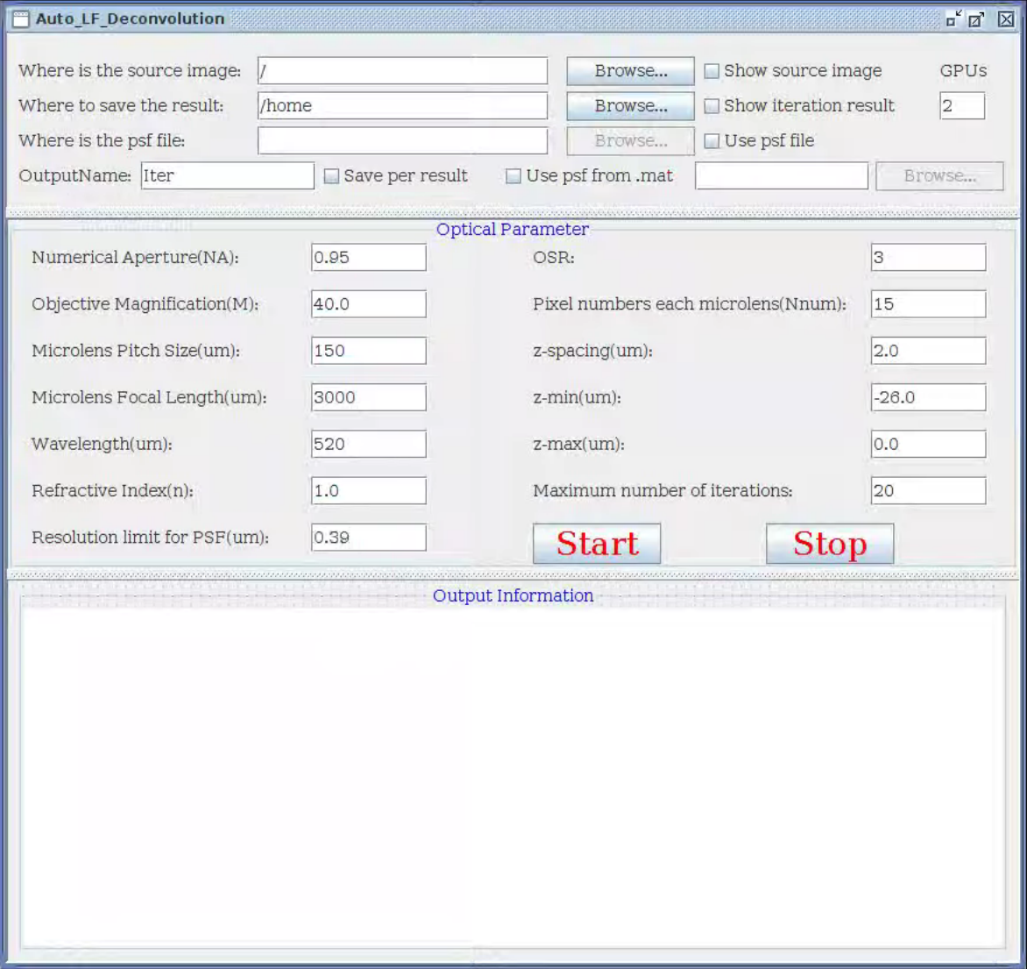


*Figure 19 Parameters settings in Auto_LF_Deconvolution*

The intermediate information about the running process will be printed in the box below called "Output information" (see fig. 20).


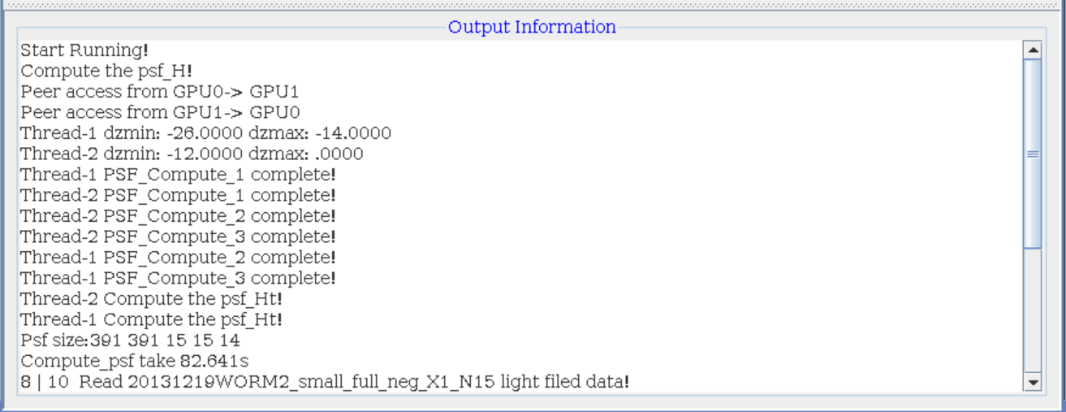


*Figure 20 Output information when running Auto_LF_Deconvolution*

The optimal iteration result predicted by the automatic prediction module will be saved under the output path.

# Experiments

We test both the MATLAB GUI program and AutoDeconJ on a server station running Ubuntu 16.04, with an Intel Core i9-9900X processor (10 physical cores and 20 threads) and 128GB RAM, equipped with four NVIDIA GEFORCE GTX 2080Ti (12G memory). The MATLAB GUI program is obtained from the supplementary material of the literature [4] and runs in the version of MATLAB R2018a. AutoDeconJ is tested using FĲI with ImageJ 1.53c.

**4.1 Less time consumption**

We demonstrate that AutoDeconJ has an excellent speed improvement in the data of C. elegans, fluorescence beads, and MCF10A cells compared to the Matlab GUI program with GPU acceleration enabled by default, including the PSF calculation and light-filed image reconstruction (see Table 1). The PSF calculation in AutoDeconJ is based on the wave optics theory and the light-field reconstruction is based on the RL algorithm, which is as same as the Matlab GUI program. The fluorescence beads data uses the following parameters: objective magnification 22.56, NA 0.5, microlens pitch size 100 um, microlens focal length 2100um, wavelength 532 um, n 1.0, OSR 3, Nnum 13, z-spacing 8um, z-max 200um, and z-min -200um. The parameters of MCF10A cells data are: objective magnification 46, NA 0.95, pitch size 97.5 um, microlens focal length 1950um, wavelength 525um, n 1.33, OSR 3, Nnum 15, z-spacing 1.4um, z-max 35um, and z-min -35um. The parameters of C. elegans data are: objective magnification 40, NA 0.8, microlens pitch size 150 um, microlens focal length 3000um, wavelength 520um, n 1.0, OSR 3, Nnum 15, z-spacing 2um, z-max 0um, and z-min -26um. The light field image size is 1635*1380 pixels for C.elegans data, 2015*2015 pixels for fluorescence beads, and 1890*1890 pixels for MCF10A cells. Compared with the Matlab GUI program, AutoDeconJ has an exponential reduction of time consumption in both the PSF calculation and the RL reconstruction, which is inversely proportional to the number of GPUs used. We have tested the data in AutodeconJ with 1, 2 and 4 Nvidia cards. The runtime of the two main functions is recorded in Table 1, where the multiplier of AutoDeconJ compared to the Matlab GUI program is shown in parentheses. In the ‘Light-field Data’ column, the square brackets after the sample name is the maximum GPU memory size required for AutoDeconJ to run. and the reconstructed volume size is below the sample name. Due to the memory limitations of a single Nvidia card, some light-field data requires more than one single GPU (such as the fluorescence beads data requires four GPUs).

Table 1. Time comparison of AutoDeconJ and MATLAB version

| Light-field Data | Function | MATLAB | AutoDeconJ  (1 GPU) | AutoDeconJ  (2 GPU) | AutoDeconJ  (4 GPU) |
| --- | --- | --- | --- | --- | --- |
| C. elegans[3.9G]  1635*1380*14 | PSF calculation | 2255.65s | 155.57s (14.5) | 85.10s (26.5) | 51.90s (44.5) |
|  | Light-field reconstruction | 18.57s | 14.06s (1.3) | 7.44s (2.5) | 4.22s (4.4) |
| MCF10A cells[26.6G]  2015*2015*51 | PSF calculation | 7999..45s | × | × | 325.82s (24.6) |
|  | Light-field reconstruction | 87.20s | × | × | 23.78s (3.7) |
| fluorescence beads[36.5G]  1890*1890*51 | PSF calculation | 20402.27s | × | × | 349.76s (58.3) |
|  | Light-field reconstruction | 83.20s | × | × | 21.79s (3.8) |

For the relationship between GPU memory size and light field data size, it is difficult to estimate only with the size of the input sample, because a large part of the memory occupation here comes from the PSF. The AutoDeconJ estimates the required memory by first calculating the PSF at the absolute farthest defocus position and then estimating the PSF size at other locations based on that. Take the data of MCF10A as an example. We calculate the PSF size at the absolute farthest defocus position, which is 511*511*15*15, and estimate the size of the whole PSF stack to be 511*511*15*15*51. And volume size to be reconstructed is 1890*1890*51. Since the reconstruction process involves forward and backward propagation, the reconstruction process requires PSF and transpose of PSF respectively for the forward and backward propagation. The final estimated memory size can be:

The function is used to get the number of bytes of the variable type. Factor 1.15 is used to cover the GPU memory occupied by temporary variables during runtime. The default unit of is megabyte (MB). We can get the final memory requirement of 27104MB for MCF10A data by calculating the , so for a GPU with 12G memory, a minimum of three are required.

**4.2 Automatic iterative prediction**

We further demonstrate the performance of the automated iterative prediction on the fluorescent beads and MCF10A cells. We totally perform 50 RL deconvolution iterations for fluorescent beads and MCF10A cells. We find that the resolution in the MIP (max intensity projection) of the x-y plane has a significant improvement with the increase of iteration numbers, but the artifacts are also substantially increased (see fig. 21a, e). We exhibit the MIP images along the z-axis for the reconstructed results with 1, 20, and the prediction number iterations respectively by AutoDeconJ in figure 20, where subregions of x-y MIP are selected to demonstrate the resolution improvement and the artifacts increase (see fig. 21a, e, b, f). For the image quality metric of the light-field deconvolution, we calculate the DCT entropy of the whole DCT (WDCT) spectrum of the x-y MIP images with the corresponding iterative numbers(see fig. 21c, g), which is marked by the white numbers (see fig. 21c, g) and named WDCT entropy. If not specified, the DCT entropy in the following refers to the triangular region. As the number of iterations increases, the overall amount of information recovered by the reconstruction is increased. But when it exceeds the resolution information limited by the PSF (which depends on the "Resolution limit for PSF", and specifically, 10.1um for MCF10A cells and 16.9um for fluorescent beads) marked in the red triangle in the upper left corner (see fig. 21c, d), the additional increase will appear as a replication of the spectrum. We thus calculate the DCT entropy of the DCT spectrum inside the red triangle (i.e., the spectrum limited by the PSF) with different iteration numbers (1~50) and plot the DCT entropy normalized curves of the x-y MIP images in the fluorescent beads and MCF10A cells data (see fig. 21d, h). We find that the 11th iterations achieve the maximum value of the DCT entropy in the results of fluorescent beads, which is within the empirically optimal range of 5~15 iterations (see fig. 21d). Similarly, the 5th iterations correspond to the maximum value of the DCT entropy in the MCF10A cell, which is within the empirically optimal range of 5~10 iterations (see fig. 21h). As such, AutoDeconJ can be used as a strong reference for the iterative reconstruction of new light field data.

**
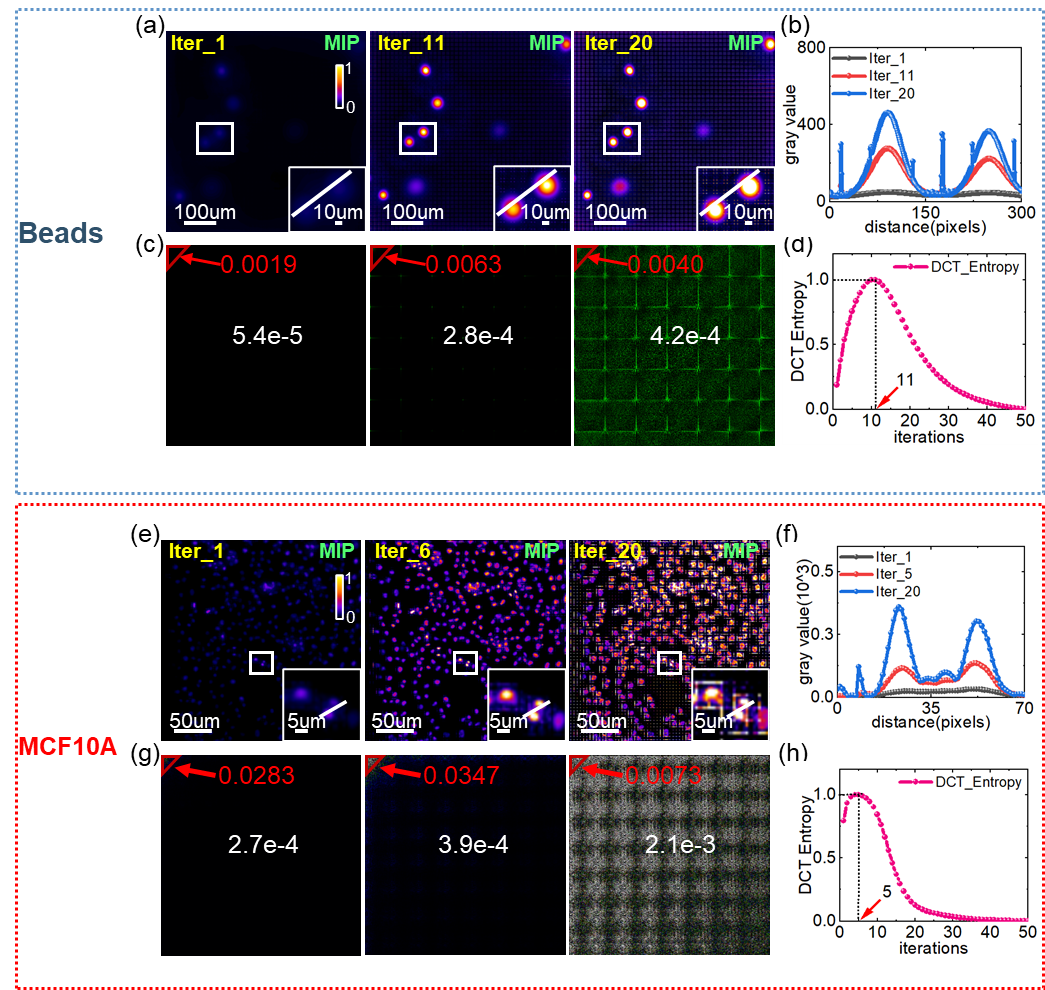
**

*Figure 21 Performance of the image quality metric. The source light-field data is fluorescence beads data and MCF10A cells data.* ***(a,e)*** *The maximum intensity projection (MIP) along the z-axis for each result with the RL decon-volution at 1, 20, and the prediction number by AutoDeconJ. The image in the lower-right corner is a magnification of the white area.* ***(b,f)*** *The gray value along solid lines in the corresponding lower-right corner of the diagram in (a,e).* ***(c,g)*** *The DCT transform corresponds to the deconvolution result in (a,e). The coefficients with higher values are shown in light-gray, and those with lower values are shown in dark-gray. The red numbers are the DCT entropy values of the red triangle region in the upper left corner, and the white numbers indicate the overall DCT entropy value. By visual comparison of the red region as well as the values, it can be observed that with the increase of the iterations, the entropy value in the low-frequency region circled in red will increase first and then become smaller again, but the overall entropy keeps increasing.* ***(d,h)****Normalized curves of DCT entropy values with the number of iterations* *for the red triangle region in (c,g), where the maximum DCT entropy value corresponds to the optimal number of iterations predicted by AutoDeconJ, which is consistent with the empirically optimal value.*

**4.3 Comparison with VCD-Net network**

We further demonstrate the superior generality of AutoDeconJ compared to the state-of-the-art VCD-Net network on the simulation data. The original high-resolution three-dimensional stack image is the BBBC024vl image set from the Broad Bioimage Benchmark Collection *(*[*https://bbbc.broadinstitute.org/BBBC024*](https://bbbc.broadinstitute.org/BBBC024)*)*, which is then processed according to the VCD-Net network data preprocessing tutorial provided by Wang, Z. et al. The PSF parameters are chosen to be the same as the tubulin data provided by Wang, Z. et al: objective magnification 40, NA 0.8, pitch size 150 um, microlens focal length 3500um, wavelength 580um, n 1.33, OSR 3, Nnum 11, z-spacing 1um, z-max 30um, and z-min -30um. We use the existing model trained for the tubulin data (by Wang, Z. et al.) to reconstruct the simulated new HL60 cell nuclei data to test the generality of VCD-Net network. We also test the simulated light field data using the proposed AutoDeconJ. A certain layer of reconstruction results and its corresponding ground-truth images are shown in figure 22. Even if all the optical parameters of the simulated HL60 cell nuclei data are the same as the tubulin data, it still gets an unacceptable result when using the trained model of the VCD-Net network for tubulin data to reconstruct the new simulation data whose source data is one of the 3D image stacks in HL60 cell nuclei sets. On the contrary, AutoDeconJ can still achieve a good result. This means that the trained model of the VCD-Net network can only reconstruct a certain class of data, having a very limited generalized capability. Even if all the parameters are the same, the model still needs to be retrained when the sample to be reconstructed changes. Furthermore, due to the limited fitting ability of the neural network, the VCD-Net network also cannot obtain satisfactory reconstructions in scenes with different microlens parameters (e.g., a different pitch size) or with samples having complex structural information.

**
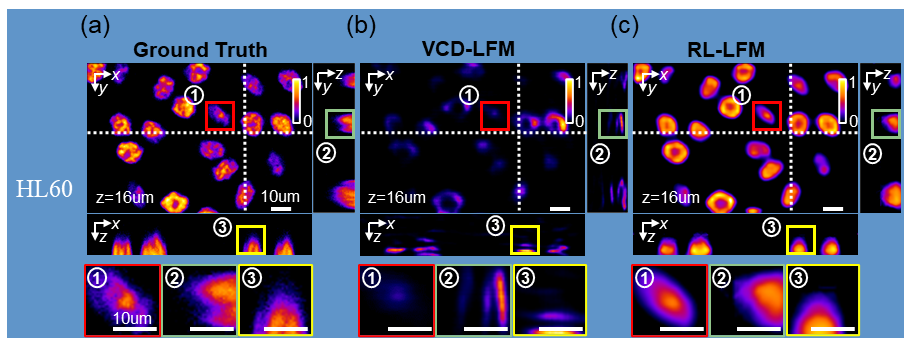
**

*Figure 22 Comparison of VCD-Net network and AutoDeconJ in generality at the simulation data of HL60 cell nuclei. The bottom is enlarged views inside the boxes (marked with a number) from different slice images on the top. The top is the x-y slice image at z=16um, with certain y-z and x-z slices marked by white dash lines.* ***(a)*** *The ground truth at z = 16um.* ***(b)*** *Reconstructed result at z = 16um by the VCD-Net shows a lot of information loss compared to the ground truth. (c) Reconstructed result at z = 16um by AutoDeconJ matches well with the ground truth.*

**4.4 Extension to other deconvolution algorithms**

We further demonstrate the performance of our iterative criterion on data acquired with wide-field microscopy. We perform deconvolution on C. elegans embryo data captured by a standard wide-field microscopy with the algorithms integrated into DeconvolutionLab2[7], including Landweber(LW), Richardson–Lucy(RL) and Richardson–Lucy with total-variation regularization(RL-TV). The software DeconvolutionLab2 and the C. elegans embryo data are available in reference [7]. We totally perform 500 RL iterations, 500 RL-TV iterations and 3000 LW iterations. The results of each iteration are energy normalized before the evaluation of the image metric. In the algorithm of the RL and RL-TV, we find that the resolution in the MIP (max intensity projection) of the x-y plane has a significant improvement with an appropriate of iteration numbers, however, beyond this number of iterations, the resolution decreases along with the increase in background signals and artefacts (see fig. 23a, e). We exhibit the MIP images along the z-axis for the reconstructed results with 1, 500, and the prediction number iterations respectively, where subregions of x-y MIP are selected to demonstrate that excessive iteration leads to a decrease in resolution and an increase in background signals (see fig. 23a, b, e, f). In the algorithm of the LW, we also find that the resolution in the MIP (max intensity projection) of the x-y plane has a significant improvement with the increase of the deconvolution (see fig. 23i). We exhibit the MIP images along the z-axis for the reconstructed results with 1, 200(suggested in DeconvolutionLab2), and the prediction number iterations respectively, where subregions of x-y MIP are selected to demonstrate that the result of the prediction iteration has a better resolution enhancement than the 200 iterations given by DeconvolutionLab2 (see fig. 23i, j). For the image quality metric of the deconvolution, we calculate the DCT entropy of the whole DCT (WDCT) spectrum of the x-y MIP images with the corresponding iterative number, which is marked by the white numbers (see fig. 23c, g, k). The image quality metric we also named WDCT entropy. If not specified, the DCT entropy in the following refers to the DCT entropy of the triangular region. With an appropriate increase in the number of iterations, the aliased signal in the low and high frequency regions is rearranged and the energy in the cutoff frequency range will increase. The cutoff frequency is determined by the resolution limitation of the microscope system PSF, which is:

where is the numerical aperture of the objective lens and is the wavelength of emission light. We thus calculate the DCT entropy of the DCT spectrum inside the red triangle (i.e., the spectrum limited by the PSF) with different iteration numbers (1~500) and plot the DCT entropy normalized curves of the x-y MIP images in the C. elegans embryo data (see fig. 23d, h, l). We find that 130th iterations achieve the maximum value of the DCT entropy in the algorithm of RL (see fig. 23d) and 136th iterations correspond to the maximum value of the DCT entropy in the algorithm of RL-TV (see fig. 23h), which are close to the 200 iterations showed in DeconvolutionLab2. Similarly, we find that 1799th iterations achieve the maximum value of the DCT entropy in the algorithm of LW (see fig. 23l). Compared to the algorithms of RL and RL-TV, the increase in artefacts and the decrease in resolution are not significant with increasing iterations in the algorithm of LW, and even an additional 1000 iterations do not result in a significant increase in artefacts and the decrease in resolution. The result shows our iterative criterion can be extended to the RL, RL-TV and LW deconvolution algorithm on wide-field microscopy.


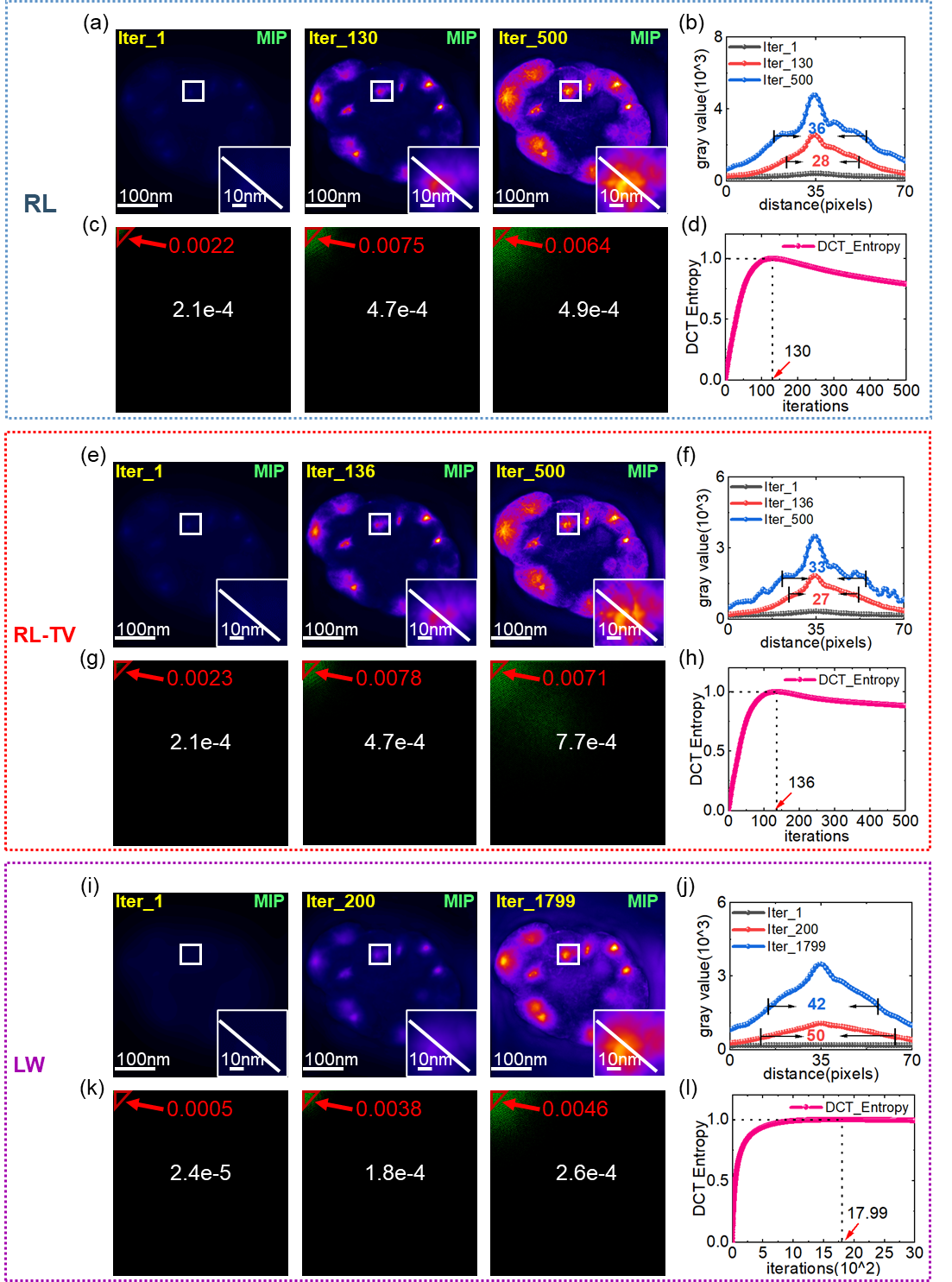


*Fig. 23 Performance of the image quality metric in wide-field microscopy data. The source wide-field microscopy data is obtained from the open-source dataset provided in DeconvolutionLib2.* ***(a)****The maximum intensity projection (MIP) along the z-axis for each result with the RL deconvolution algorithm at 1, 500, and the prediction number by our iteration criterion. The image in the lower-right corner is a magnification of the white area.* ***(b)*** *The gray value along solid lines in the lower-right corner of the diagram in (a) and the colour numbers correspond to the full width at half maximum of the curve.* ***(c)*** *The DCT transform corresponding to the deconvolution result in (a). The coefficients with higher values are shown in light-gray, and those with lower values are shown in dark-gray. The red numbers are the DCT entropy values of the red triangle in the upper left corner, and the white numbers indicate the overall DCT entropy value.* ***(d)*** *The normalized curve of DCT entropy values with the number of iterations for the red region in (c), where the maximum DCT entropy value corresponds to the optimal number of 130th iterations predicted by our iteration criterion.* ***(e)****The maximum intensity projection (MIP) along the z-axis for each result with the RL-TV deconvolution algorithm at 1, 500, and the prediction number by our iteration criterion. The image in the lower-right corner is a magnification of the white area.* ***(f)*** *The gray value along solid lines in the lower-right corner of the diagram in (e) and the colour numbers correspond to the full width at half maximum of the curve.* ***(g)*** *The DCT transform corresponding to the deconvolution result in (e). The coefficients with higher values are shown in light-gray, and those with lower values are shown in dark-gray. The red numbers are the DCT entropy values of the red triangle in the upper left corner, and the white numbers indicate the overall DCT entropy value.* ***(h)*** *The normalized curve of DCT entropy values with the number of iterations for the red region in (g), where the maximum DCT entropy value corresponds to the optimal number of 136th iterations predicted by our iteration criterion.* ***(i)*** *The maximum intensity projection (MIP) along the z-axis for each result with the LW deconvolution algorithm at 1, 200 (given by DeconvolutionLab2), and the prediction number by our iteration criterion. The image in the lower-right corner is a magnification of the white area.* ***(j)*** *The gray value along solid lines in the lower-right corner of the diagram in (i) and the colour numbers correspond to the full width at half maximum of the curve.* ***(k)*** *The DCT transform corresponding to the deconvolution result in (i). The coefficients with higher values are shown in light-gray, and those with lower values are shown in dark-gray. The red numbers are the DCT entropy values of the red triangle in the upper left corner, and the white numbers indicate the overall DCT entropy value.* ***(l)*** *The normalized curve of DCT entropy values with the number of iterations for the red region in (k), where the maximum DCT entropy value corresponds to the optimal number of 1799th iterations predicted by our iteration criterion.*

References

1 Schindelin J, Rueden C T, Hiner M C, et al. The ImageJ ecosystem: An open platform for biomedical image analysis[J]. Molecular reproduction and development, 2015, 82(7-8): 518-529.

2 Schindelin J, Arganda-Carreras I, Frise E, et al. Fiji: an open-source platform for biological-image analysis[J]. Nature methods, 2012, 9(7): 676-682.

3 Yan Y, Grossman M, Sarkar V. JCUDA: A programmer-friendly interface for accelerating Java programs with CUDA[C]//European Conference on Parallel Processing. Springer, Berlin, Heidelberg, 2009: 887-899.

4 Prevedel R, Yoon Y G, Hoffmann M, et al. Simultaneous whole-animal 3D imaging of neuronal activity using light-field microscopy[J]. Nature methods, 2014, 11(7): 727-730.

5 Svoboda D, Kozubek M, Stejskal S. Generation of digital phantoms of cell nuclei and simulation of image formation in 3D image cytometry[J]. Cytometry Part A: The Journal of the International Society for Advancement of Cytometry, 2009, 75(6): 494-509.

6 Wang Z, Zhu L, Zhang H, et al. Real-time volumetric reconstruction of biological dynamics with light-field microscopy and deep learning[J]. Nature Methods, 2021, 18(5): 551-556.

7 Sage D, Donati L, Soulez F, et al. DeconvolutionLab2: An open-source software for deconvolution microscopy[J]. Methods, 2017, 115: 28-41.
